# Beyond Return to Work: The Effect of Multimorbidity on Work Functioning Trajectories After Sick Leave due to Common Mental Disorders

**DOI:** 10.1007/s10926-016-9647-0

**Published:** 2016-06-01

**Authors:** Monica Ubalde-Lopez, I. Arends, J. Almansa, G. L. Delclos, D. Gimeno, U. Bültmann

**Affiliations:** 1 CISAL-Center for Research in Occupational Health, Barcelona Biomedical Research Park (PRBB), Pompeu Fabra University, C/Dr. Aiguader, 80, 08003 Barcelona, Spain; 2 CIBERESP, CIBER in Epidemiology and Public Health, Madrid, Spain; 3 IMIM (Hospital del Mar Medical Research Institute), Barcelona, Spain; 4 Department Tranzo, Tilburg School of Social and Behavioral Sciences, Tilburg University, Tilburg, The Netherlands; 5 Department of Health Sciences, Community and Occupational Medicine, University Medical Center Groningen, University of Groningen, Groningen, The Netherlands; 6 Southwest Center for Occupational and Environmental Health, Division of Epidemiology, Human Genetics, and Environmental Sciences, The University of Texas Health Science Center at Houston, Houston, TX USA; 7 Southwest Center for Occupational and Environmental Health, Division of Epidemiology, Human Genetics, and Environmental Sciences, The University of Texas School of Public Health, San Antonio Campus, San Antonio, TX USA

**Keywords:** Mental health, Chronic health conditions, Job performance, Work capacity, Sickness absence

## Abstract

*Objectives* Patients with common mental disorders (CMDs) often suffer from comorbidities, which may limit their functioning at work. We assessed the longitudinal impact of multimorbidity, defined as two or more co-occurring chronic health conditions, on work functioning over time among workers who had returned to work after sick leave due to CMDs. *Methods* Prospective cohort study of 156 workers followed for 1 year after return to work from sick leave due to CMDs. A multimorbidity score was computed by counting severity-weighted chronic health conditions measured at baseline. Work functioning was measured at baseline and at 3, 6 and 12 months follow-up with the Work Role Functioning Questionnaire. Work functioning trajectories, i.e. the course of work functioning after return to work over time, were identified through latent class growth analysis. *Results* A total of 44 % of workers had multimorbidity. Four work functioning trajectories were identified: one (12 % of the workers) showed increasing work functioning scores during follow-up, whereas the other trajectories showed low, medium and high scores (23, 41 and 25 %, respectively) that remained stable across time points. Although multimorbidity did not predict membership in any trajectory, within the increasing score trajectory levels of work functioning were lower among those with high baseline multimorbidity score (*p* < 0.001). *Conclusions* Over time, multimorbidity negatively impacts work functioning after return to work from sick leave due to CMDs.

## Introduction

Common mental disorders (CMDs) are a major social and economic problem among working populations, because of their high prevalence [1], impact on work functioning [2] and consequences in terms of long-term and recurrent sickness absence [3, 4], and early retirement [5, 6]. As such, return to work (RTW) after sick leave for a common mental disorder (CMD) has been extensively studied. Various factors predict RTW among workers with CMDs, including severity of the mental health problem, time until seeking help while on sick leave [7], self-expectations on return to work and prior sickness absence due to CMDs [8].

Although different interventions have been developed to facilitate RTW [9, 10] and reduce recurrent sick leave after RTW among workers with CMDs [11], the course of work functioning over time after RTW has not been assessed [12]. Workers who return to work after CMDs may still struggle with health-related work limitations that limit their ability to meet work demands, resulting in reduced job performance [13, 14] and productivity at work [15]. According to the Organization for Economic Co-operation and Development (OECD), reduced productivity at work is reported among three in four workers with mental disorders across Western countries, compared to one in four among those without [16]. Individual work functioning is reflected by the balance between job demands and a given health state, varying from working successfully in a productive and healthy way to being absent from work [17]. Serially assessing workers’ self-perceived health-related work functioning, using instruments like the Work Role Functioning Questionnaire after RTW, may help identify workers who need support to stay at work and to develop appropriate interventions.

Chronic health conditions limit the ability to carry out specific work demands, and to function at the workplace. Previous studies have shown that the number of chronic health conditions increases the risk of physical and psychosocial work limitations [18]. Moreover, there is an incremental effect across combinations of the number and type of chronic health conditions on predicting sick leave and work-related impairment [19]. A growing body of literature has shown the negative impact of chronic health conditions, not only on work functioning but also on staying at work, sickness absence and work disability. However, less is known about whether chronic diseases impact the course of those indicators over time. To our knowledge, the effect of multimorbidity, defined as the co-occurrence of two or more chronic or acute health conditions considering none as the primary [20], on work functioning after RTW has not yet been examined. We assessed the impact, over 12 months, of baseline multimorbidity on work functioning trajectories in workers who had returned to work after a CMD-related sick leave, based on four measurement waves.

## Methods

### Study Design and Participants

This was a prospective cohort study of employees who returned to work after a sick leave episode due to a CMD. Employees were followed-up at 3, 6 and 12 months after RTW. Participants (n = 156) were recruited between January 2010 and June 2011 as part of a cluster-randomized controlled trial (cluster-RCT) study (“SHARP-at work”) focused on the implementation and evaluation of an at-work intervention to prevent recurrent sickness absence after sick leave for a CMD [11]. Recruitment was carried out by occupational physicians (OPs) from one of the largest occupational health services in the Netherlands. Inclusion criteria for the cluster-RTC were: (a) 18–63 years of age, (b) employed in a paid job; (c) a CMD (consisting of depressive, anxiety and adjustment disorders) diagnosed by the OP at the start of the sick leave episode; (d) sick leave duration between 2 weeks and 12 months and (e) a planned RTW within 2 weeks. Diagnoses were coded by OPs according to the Classification of Diseases in Dutch [21], based on the ICD-10 International Classification of Diseases [22]. Exclusion criteria were: (a) having had a prior CMD-related sick leave in the 3 months prior to the present episode; (b) previous diagnosis of a psychotic, bipolar or post-traumatic stress disorder; (c) reporting somatic complaints that commonly influence work ability; (d) pregnancy, upcoming retirement, resignation or firing; and (e) inability to communicate in Dutch. Eligible employees were asked to participate in the study and provided written informed consent. The study protocol was reviewed and approved by the Medical Ethics Board of the University Medical Center Groningen.

### Measurements

#### Work Functioning

Work functioning was measured at baseline and 3, 6 and 12 months after RTW, using the cross-culturally adapted, translated and validated Dutch version of the Work Role Functioning Questionnaire (WRFQ) [23, 24]. The WRFQ measures perceived limitations in meeting work demands due to physical or emotional problems [17, 25]. The instrument consists of 27 items in five subdomains [24]: (1) work scheduling demands; (2) output demands; (3) physical demands; (4) mental demands; and (5) social demands. Response options vary from 0 % (none of the time), 50 % (half of the time) to 100 % (all of the time). Scores were converted to a 0–100 score scale, with higher scores indicating better work functioning.

#### Multimorbidity

Multimorbidity was defined as having two or more chronic health conditions. Chronic health conditions were self-reported at baseline, in response to a list of 13 system-specific diagnosis groups: injuries, musculoskeletal, mental, cardiovascular, respiratory, neurological, digestive, urogenital, skin, endocrine/metabolism, blood and congenital diseases and tumors. Self-reported weight and height were used to calculate body mass index (BMI); obesity was defined as a BMI ≥ 30 kg/m^2^ [26].

To measure multimorbidity we first examined the severity of each diagnosis group, by assessing their impact on poor general health status, as an indicator of health-related quality of life [27–30]. General health status was measured through a self-reported single item question of the 36-item Short-Form Health Survey (SF-36): “In general, how would you rate your health?” Responses were dichotomized as good (excellent, very good, good) or poor (fair and poor). Next, we weighted self-reported chronic health conditions as severity scores related to their impact on poor general health [27, 28]: from 1 = low (i.e., CVD and metabolism diseases); and 2 = intermediate (i.e., obesity, respiratory and tumors) to 3 = high (i.e., mental, musculoskeletal disorders, skin neurological and digestive diseases). In the final step, a multimorbidity score (MMBS) was calculated by adding severity scores of workers who reported two or more chronic health conditions. Participants reporting fewer than two chronic health conditions were coded as MMBS = 0 (i.e., without multimorbidity). All workers had been diagnosed with a mental disorder. As such, any self-reported chronic health condition (other than mental disorder) was regarded as a second chronic health condition (i.e., with multimorbidity). Chronic conditions for which there were three or fewer cases (i.e., injuries, urogenital, blood and congenital diseases) were excluded from the analysis.

### Covariates

Demographic factors such as age, sex and education level (i.e., low, medium, high), intervention group (i.e., intervention, usual care), and health-related behaviors such as physical activity (i.e., never or one time, two to seven times and more than seven times of 1/2 h of physical activities/week), alcohol (i.e., 1–4, 15–21, 22–34, 35–50 and >50 glasses of alcohol/week) and smoking (no, not anymore, yes) were measured at baseline using a questionnaire (electronic or paper version), that was sent to participants, after securing informed consent.

### Statistical Analysis

Characteristics of the study population were described at baseline, summarizing categorical variables as frequencies and continuous variables using central tendency measures. The work functioning score was expressed as an overall mean score at baseline, 3, 6 and 12 months.

To measure the impact of each self-reported chronic condition on poor general health status, we fit logistic regressions adjusting for age, sex and health-related behaviors. Severity scores for diagnosis group ranged from 1 to 3, as mentioned above, and were based on the magnitude of the adjusted odds ratios (AORs) obtained as follows: 1 for AORs from 0 to 1, 2 for AORs from >1 to 2 and 3 for AORs over 2.

Trajectories of work functioning scores were identified based on all four measurement waves using latent class growth analysis (LCGA). LCGA identifies differentiated subpopulations (latent classes), each with its own specific longitudinal trend [31]. We used an unstructured time (discrete) trend, so there was no a priori assumption about the shape structure in which work functioning scores evolve over time. Specifically, the trajectories were predicted by including time as a categorical variable with dummy coding. The parameters related to the time variable provide the expected mean of work functioning per time-point. Trajectories were adjusted for the MMBS, entered in the model as a continuous variable, as a class-trajectory modifier within each trajectory, assuming a constant effect over time. The trajectories were also adjusted for age, sex, educational level and intervention group (equal effect across classes). The MMBS was also included as trajectory class membership predictor. Each latent class has specific time trajectory parameters defining its expected trajectory, as well as different residual variances. To set the optimal number of classes, the Bayesian information criterion (BIC) was used. BIC is based on the number of parameters in the model and the log-likelihood of the model; the optimum model is the one with the lowest BIC [32]. To avoid local maxima solution the model was run with 150 different starting values.

The LCGA also provides an estimation of class membership probabilities for each individual. These probabilities were used in order to investigate the relationship between class-trajectories and baseline characteristics. Differences in baseline characteristics on MMBS, sex, age, educational level and intervention group were tested by Pearson’s Chi square tests for categorical variables (crosstabs weighted by membership probability) and (weighted) means for the continuous one. The statistical analyses were conducted using LatentGold 4.5 for LCGA, and SPSS V.19.0 for descriptive analyses.

## Results

At baseline our study population consisted of 156 adults (60 % female) with a mean age of 42 (SD = 9.6) years, mostly medium (49 %) and highly educated (39 %). Other health-related characteristics are shown in Table 1. Fifty-eight percent of workers reported at least one chronic health condition and 44 % had multimorbidity. The most common self-reported chronic health conditions were musculoskeletal disorders (24 %), cardiovascular diseases (13 %) and obesity (12 %). Digestive, skin and neurological diseases, as well as mental and musculoskeletal disorders showed the strongest impact on poor general health (AOR ≥ 2) (“Appendix”). Among those with multimorbidity, the MMBS ranged from 2 to 14 with a mean score of 7.2 (SD 2.6). Participants at 3, 6 and 12 months measurement waves were: 136 (87 %), 120 (77 %) and 105 (67 %) respectively.

**Table 1:** Population baseline characteristics (N = 156)

Variables	N	%
*Gender (female)*	91	58.6
*Educational level*		
Low	19	12.2
Medium	75	48.7
High	61	39.1
*Group (intervention)*	80	51.3
*Chronic conditions*		
Mental disorder (MD)	110	70.1
MD + other	65	41.6
Musculoskeletal	38	24.2
Cardiovascular	20	12.7
Obesity	19	12.1
Skin	16	10.2
Neurological	15	9.6
Respiratory	13	8.3
Digestive	13	8.3
Metabolism	7	4.5
Urogenital	3	1.9
Tumors	3	1.9
Blood	2	1.3
Injuries	1	0.6
Congenital	1	0.6
Others	11	7.0
*Health related behaviors*		
*Alcohol* ^a^		
None	56	35.7
1–4	91	58.0
15–21	7	4.5
22–34	2	1.3
35–50	1	0.6
>50	0	0.0
*Smoking*		
No	91	58.0
Not anymore	29	18.5
Yes	37	23.6
*Physical activity* ^b^		
Never or one time	25	15.9
2–7 times	119	75.8
More than seven times	13	8.3
*General health*		
Good	110	71.0
Poor	45	29.0

^a^Glasses of alcohol/week, ^b^ 1/2 h of physical activities/week

**Fig. 1:**
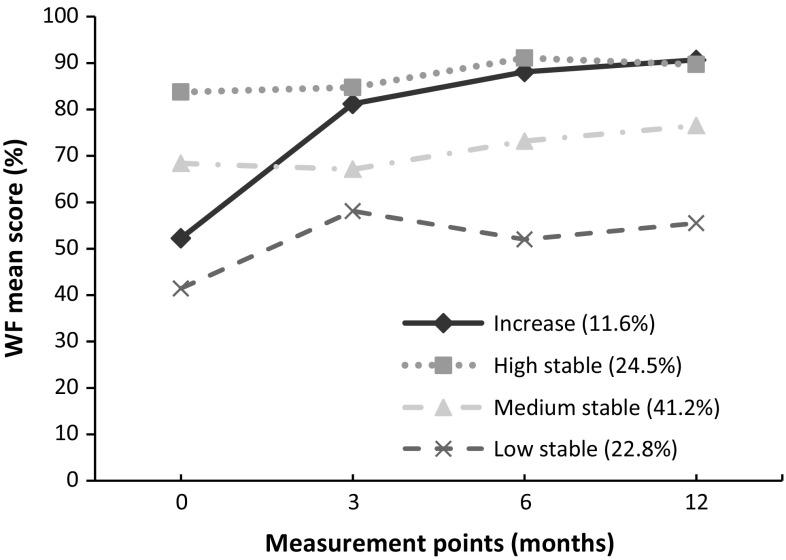
Trajectories of work functioning scores after return to work from a sick leave due to a common mental disorder

Figure 1 shows that the LCGA revealed four trajectories for work functioning scores: one with increasing work functioning scores during follow-up (12 % of the workers), while the other three showed low stable (23 %), medium stable (41 %) and high stable work functioning scores (25 %). Multimorbidity did not influence the likelihood of belonging to one of the four trajectories (*p* value = 0.24), although workers within the high stable work functioning trajectory tended to have no multimorbidity compared to the stable trajectories (70 and ±50 % respectively), and those within the medium and low stable had higher MMBS (mean ± SD; 7.4 ± 2.8 and 7.7 ± 2.2 respectively). No significant differences were found across trajectories for further population characteristics, except that those within the low stable trajectory were significantly more likely to belong to the control group (usual care) of the intervention study in contrast to the other three trajectories (70 and ±40 % respectively, *p* value = 0.03) (Table 2).

**Table 2:** Distribution of class membership probabilities, expected numbers and percentages across work functioning trajectories

Variables	WF trajectories								Total N	*p* value
	Increment		High stable		Medium stable		Low stable			
*MMB score*										0.51
Mean (SD)	6.5 (3.3)		6.4 (1.5)		7.4 (2.8)		7.7 (2.2)		68	

	N	%	N	%	N	%	N	%		
*MMB*										0.24
No MMB	9.5	52.8	26.7	70.3	31.9	50.1	18.9	53.5	88	
MMB	8.5	47.2	11.3	29.7	31.8	49.9	16.4	46.5	68	
*Age*										0.90
≤44	9.1	50.8	23.8	62.7	39.8	62.4	18.3	51.8	92	
45–54	5.6	31.4	10.6	28.0	15.8	24.7	11	31.1	43	
≥55	3.2	17.8	3.5	9.3	8.2	12.9	6	17.1	21	
*Sex*										0.84
Male	6.3	34.8	16.3	43.0	25.1	39.3	16.3	46.3	65	
Female	11.7	65.2	21.6	57.0	38.7	60.7	18.9	53.7	91	
*Educational level*										0.99
Low	3.1	17.4	4.3	11.3	7.6	11.9	4	11.3	19	
Medium	7.3	40.7	19	50.0	30.5	47.5	18.4	52.1	75	
High	7.5	41.8	14.7	38.7	25.9	40.6	12.9	36.6	61	
*Intervention group*										0.03
Control	7.3	40.4	15.9	41.8	27	42.3	24.9	70.5	76	
Intervention	10.7	59.6	22.1	58.2	36.8	57.7	10.4	29.5	80	
*TOTAL*	17.9	11.6	37.9	25.1	63.8	40.3	35.3	22.8	156	

*MMB* multimorbidity, *WF* work functioning, *SD* standard deviation

As for the effect of MMBS as a modifier of trajectories over time, the LCGA showed that within the increasing work functioning scores trajectory, work functioning decreased over time with higher baseline MMBS. Each unit increase in baseline MMBS implied a reduction of 1.4 points (*p* value <0.001) of the work functioning score over time. No significant effect was observed within the other three trajectories, although work functioning trajectories with lower values (Table 3) tended to show higher MMBS (Table 2).

**Table 3:** Effect of MMBS on work functioning trajectories after return to work from sick leave due to a common mental disorder

Class-trajectories	WF coefficient	*p* value	Wald test *p* value
Increase	−1.37	0.000	0.000
High stable	0.26	0.450	
Medium stable	−0.12	0.710	
Low stable	−0.20	0.580	

*MMBS* multimorbidity score, *WF* work functioning

## Discussion

To our knowledge, this is the first study to analyze work functioning trajectories, and the effect multimorbidity may have on these trajectories, after return to work following a sick leave episode due to a CMD.

Overall, the prevalence of multimorbidity in this study population was high, at 44 %, compared to multimorbidity rates (35 %) among the Dutch general population aged 50 years or older [33]. This is not surprising because many other morbidities have been linked to mental disorders. Almost 80 % of CMD patients have at least one other co-occurring health-related condition, and co-existing physical health problems are known as predictors for the onset and persistence of CMDs [34]. As observed in previous studies, we found musculoskeletal disorders to be the most common self-reported chronic condition that had the greatest impact on poor health [28, 29] together with mental disorders, skin, neurological and digestive diseases. We identified four different groups of workers that followed similar work functioning trajectories in the year following return to work. Three of these trajectories were stable over the course of the follow-up period, but one showed increasing work functioning scores. The usefulness of applying group-based trajectory modeling methodology to examine occupational outcomes, such as work functioning, is that it allows tracking how these outcomes evolve over time from an individual-centered rather than variable-centered perspective. This enables identification of the percentages of individuals following different trajectories in the data [35, 36].

Although we found baseline multimorbidity did not influence the probability of belonging to a specific work functioning trajectory after RTW, most of the workers in the high stable trajectory had no multimorbidity. Only 12 % of workers improved their work functioning over the 12-month follow-up, while the remainder was stable. One possible explanation for this finding might be that workers who maintained low work functioning scores during follow-up (i.e., from 40 to 50 % of the time with difficulties meeting work demands) largely belonged to the control (“usual care”) group (70 %). Finally, a worrying finding is that only few workers (24 %) belong to the high stable trajectory.

We found that baseline multimorbidity was related to lower work functioning scores after return to work. A significant association was only found in the trajectory where work functioning scores increased during the follow-up period. Within this trajectory, when baseline multimorbidity was high, the subsequent improvement in work role functioning was dampened. Multimorbidity had no effect on the remaining three trajectories, all of which were stable over time. In the high stable trajectory, it could be that those workers who had better mental health were more adapted to their chronic conditions and coped with their multimorbidity without affecting work functioning. In contrast, in the low and medium stable trajectories, the lower work functioning scores may be the result of poorer mental health, to the point that multimorbidity might not have had an additional effect.

Overall, the study population had relatively high scores on some mental health measures and lower mean work functioning scores compared to a healthy population [24], despite showing some improvement over time [12]. In the Dutch occupational context, sick-listed workers cannot be dismissed and no distinction is made among work-related and non-work-related sickness absence episodes. During the first 2 years of sickness absence, at least 70 % of the wage is covered by the employer. Return to work is often a gradual process that includes the possibility of modified or partial return to work, guided by assessment and agreement among OPs, employers and employees. Both must plan for a return to work within 8 weeks after reporting sick [37]. It is conceivable that some workers returned to work with some reduction in symptoms, but still not ready to fully perform at work. Although recent studies have found early RTW beneficial on increasing work participation [38], and that symptom recovery seems not to impact RTW [39], the effect of early RTW on the relationship between remaining complaints and the ability to perform at work has not been fully addressed.

Some considerations may limit the interpretation of our findings. The sample size was somewhat small and statistical significance was not a primary aim. Rather, we were more interested in using trajectory modeling to identify meaningful relationships between coexisting chronic conditions and work functioning trajectories. Some bias may have been introduced due to loss to follow up (33 %), if lost workers were more or less likely to show the effect of multimorbidity on work functioning. The period of follow-up may have been too short to fully examine the effects of multimorbidity over time; in this regard, larger, longer prospective studies would be useful. In addition, the responsiveness for the Dutch version of the WRFQ has not yet been assessed. Thus, changes of work functioning over time might have not been fully captured. This might explain our finding of three out of four stable work functioning trajectories, although one clearly showed a change over time. Chronic health conditions were self-reported, although the predictive accuracy of self-reported morbidity has been validated in previous health interview surveys [40]. Finally, information on other workplace factors, such as organizational factors, was not available, and may have confounded or modified the effect of the associations observed. These factors could have allowed assessment, for example, of whether work-related factors were more related to health-related limitations at work than multimorbidity itself [41], either alone or clustering with the CMD diagnosis.

The main strengths of this study relate to the serial measurements of outcome during follow-up, the large geographic area in the Netherlands involved, and the variety of company sizes and economic sectors. Multimorbidity score calculations were based on severity weighted health-related conditions rather than just counting the number of conditions. In principle, this study represents a novel starting point from which one can approach the role of multiple chronic conditions on work functioning. Until now, studies analyzing the impact of chronic health conditions on different occupational outcomes have generally examined a single, specific health condition [42–44]. Few have considered the effect of multiple coexisting chronic conditions on occupational outcomes such as sickness absence and work performance [45, 46], and none have focused on the course of work functioning over time. Measuring multimorbidity may help identify workers with a CMD who return to work and still may need special attention. Successful support could conceivably help shorten time to effective reincorporation at work and improve work functioning, leading to a greater chance of meaningful work retention. The design of interventions on return to work programs, to prevent relapse and future and longer sick leave episodes after return to work, may benefit from incorporating measures to detect and better manage factors related to decreased work functioning in workers with multimorbidity.

## Appendix

See Table 4.

**Table 4:** Impact of self-reported chronic conditions on poor general health (N = 156)

Chronic conditions	N	%	OR	95 % CI	AOR	95 % CI
Mental disorders	110	70.1	3.88	1.87–8.06	3.77	1.70–8.36
Musculoskeletal	38	24.2	2.21	1.02–4.76	2.05	0.84–5.00
Cardiovascular	20	12.7	0.62	0.19–1.98	0.78	0.23–2.65
Obesity	19	12.1	1.50	0.55–4.07	1.90	0.62–5.87
Skin	16	10.2	2.07	0.72–5.94	2.36	0.75–7.41
Neurological	15	9.6	3.18	1.08–9.38	2.46	0.75–8.07
Respiratory	13	8.3	1.10	0.32–3.76	1.29	0.33–4.99
Digestive	13	8.3	5.73	1.63–21.14	13.64	3.32–56.00
Metabolism	7	4.5	0.48	0.05–4.20	0.50	0.05–4.92
Tumors	3	1.9	1.23	0.11–13.88	1.12	0.06–21.01

*OR* odds ratio, *AOR* adjusted odds ratio for health related behaviors (i.e., alcohol consumption, smoking and physical activity), *95* *% CI* 95 % confidence interval
